# The Non-structural Protein 5 and Matrix Protein Are Antigenic Targets of T Cell Immunity to Genotype 1 Porcine Reproductive and Respiratory Syndrome Viruses

**DOI:** 10.3389/fimmu.2016.00040

**Published:** 2016-02-16

**Authors:** Helen Mokhtar, Miriam Pedrera, Jean-Pierre Frossard, Lucia Biffar, Sabine E. Hammer, Lise K. Kvisgaard, Lars E. Larsen, Graham R. Stewart, Satyanarayana Somavarapu, Falko Steinbach, Simon P. Graham

**Affiliations:** ^1^Virology Department, Animal and Plant Health Agency, Addlestone, UK; ^2^Department of Microbial and Cellular Sciences, University of Surrey, Guildford, UK; ^3^Department of Pathobiology, Institute of Immunology, University of Veterinary Medicine Vienna, Vienna, Austria; ^4^National Veterinary Institute, Technical University of Denmark, Frederiksberg, Denmark; ^5^School of Pharmacy, University College London, London, UK

**Keywords:** porcine reproductive and respiratory syndrome virus, T cell, IFN-γ, antigen identification, phenotype and function, vaccine

## Abstract

The porcine reproductive and respiratory syndrome virus (PRRSV) is the cause of one of the most economically important diseases affecting swine worldwide. Efforts to develop a next-generation vaccine have largely focused on envelope glycoproteins to target virus-neutralizing antibody responses. However, these approaches have failed to demonstrate the necessary efficacy to progress toward market. T cells are crucial to the control of many viruses through cytolysis and cytokine secretion. Since control of PRRSV infection is not dependent on the development of neutralizing antibodies, it has been proposed that T cell-mediated immunity plays a key role. Therefore, we hypothesized that conserved T cell antigens represent prime candidates for the development a novel PRRS vaccine. Antigens were identified by screening a proteome-wide synthetic peptide library with T cells from cohorts of pigs rendered immune by experimental infections with a closely related (subtype 1) or divergent (subtype 3) PRRSV-1 strain. Dominant T cell IFN-γ responses were directed against the non-structural protein 5 (NSP5), and to a lesser extent, the matrix (M) protein. The majority of NSP5-specific CD8 T cells and M-specific CD4 T cells expressed a putative effector memory phenotype and were polyfunctional as assessed by coexpression of TNF-α and mobilization of the cytotoxic degranulation marker CD107a. Both antigens were generally well conserved among strains of both PRRSV genotypes. Thus, M and NSP5 represent attractive vaccine candidate T cell antigens, which should be evaluated further in the context of PRRSV vaccine development.

## Introduction

Porcine reproductive and respiratory syndrome (PRRS) is one of the most important pig diseases worldwide with a huge economic impact, estimated in the USA alone to exceed $600 million annually (1). The PRRS virus (PRRSV) is an arterivirus, which exists in two distinct genotypes: 1 (formerly European) and 2 (formerly North American). Common to most RNA viruses, the PRRSV replication cycle is prone to mutation and recombination events resulting in rapid evolution. This characteristic is most dramatically illustrated by the recent emergence of highly pathogenic variants in Southeast Asia and Eastern Europe (2–8). Efficacious control strategies that will address PRRSV variability are therefore urgently sought.

Vaccination is considered a key element to PRRS control, and both live attenuated and inactivated vaccines derived from the two PRRSV genotypes are licensed. Live attenuated vaccines are widely accepted to be the more efficacious and confer protection from disease following challenge with homologous PRRSV strains but offer variable levels of protection against heterologous strains (9). Vaccination with live vaccines can also result in long-lasting viremia and may be transmitted by viral shedding to unvaccinated pigs and transplacentally in pregnant sows leading to congenitally infected litters (10). The potential of vaccine strains to revert to virulence is a further safety concern, with vaccine-derived isolates shown to be pneumovirulent in experimentally infected pigs (11) and linked to PRRS disease outbreaks in the field (12). Reversion to virulence has been described for live vaccines of both genotypes and may occur through either mutation or recombination events (13, 14). Inactivated PRRSV vaccines are safe but are of limited efficacy, affording little to no protection against heterologous challenge (15). To date, the majority of experimental subunit approaches have focused on the envelope glycoprotein, GP5, as this had long been considered the major target of neutralizing antibodies (nAbs). However, recombinant GP5 protein was poorly immunogenic, failed to provide protection, and even exacerbated disease upon challenge (16). Expression of GP5 using plasmid DNA or viral vectors, alone or in conjunction with other structural proteins, have shown variable immunogenicity and at best confer a limited degree of protection (17–21). The limitations of existing and experimental vaccines support the investigation of novel approaches to PRRSV vaccine development.

Porcine reproductive and respiratory syndrome virus infection is associated with immunomodulation of innate immune response, which negatively impacts induction of the adaptive immune responses required for effective virus control. While PRRSV-specific antibody responses are observed from 7 to 10 days postinfection, nAb responses are often not observed until >4 weeks postinfection (22). Moreover, the level of nAbs when measurable is often far lower than those elicited by other viruses (23). Since animals may clear PRRSV infection and be immune to reinfection in the absence of nAbs, cell-mediated responses are thought to play an important role (5, 6, 23, 24). PRRSV infection results in virus-specific T cells detectable in the blood after 7–14 days, and we recently showed that a pathogenic PRRSV-1 subtype 3 strain induced a stronger IFN-γ response than conventional strains, which was associated with enhanced clearance of the virus (5). Few studies have conducted an in-depth analysis of the PRRSV-specific T cell response. While an early study found that CD4 T cells were necessary to drive PRRSV-specific proliferative responses *in vitro* (25), a more recent study indicated that CD8 T cells are the predominant population expanded by *in vitro* PRRSV stimulation (26). We have shown that both CD4 and CD8 T cells contribute to PRRSV-specific IFN-γ responses (27). While IFN-γ is known to inhibit PRRSV replication at least *in vitro* (28, 29), cytotoxic killing of infected cells by CD8 T cells likely represents an important effector mechanism *in vivo* (30), and CD8 T cells are the dominant T cell population infiltrating the lungs during PRRSV infection (31). With regards to T cell specificity, we previously reported a range of IFN-γ reactivity to PRRSV-1 proteins, most notably to the M protein, as well as the viral polymerase, NSPs 1, 2, and 5, and GP5 (27), many of which had also been described by others to be T cell antigens (32–37).

Therefore, we hypothesize that conserved PRRSV antigens that are the targets of T cell responses represent prime candidates for the development of a novel PRRS vaccine. To address this, an attenuated subtype 1 and a pathogenic subtype 3 PRRSV-1 strain were used in an experimental infection and challenge model. T cell reactivity was monitored longitudinally and antigen reactivity assessed after each infection by screening of a proteome-wide synthetic PRRSV peptide library. Two antigens that were strongly recognized by both groups of animals were selected for detailed study. Flow cytometric analyses quantitatively and qualitatively defined the specificity, phenotype, and function of antigen-specific T cells.

## Materials and Methods

### Viruses

The PRRSV-1 subtype 1 MARC-145 cell attenuated Olot/91 strain was kindly provided by Dr. Sonia Zúñiga and Prof. Luis Enjuanes, Centro Nacional de Biotecnología, Madrid, Spain, and propagated in MARC-145 cells (27). The virulent PRRSV-1 subtype 3 strain SU1-Bel (isolated from material kindly provided by Dr. Tomasz Stadejek, Warsaw University of Life Sciences, Poland) and the PRRSV-1 subtype 1 strain 215-06 were both propagated in porcine alveolar macrophages [PAMs; Cell and Tissue Culture Unit, Animal and Plant Health Agency (APHA), Addlestone, UK] (5).

### PRRSV-1 Peptides and Proteins

A synthetic overlapping peptide library of 1275 pentadecamer peptides off-set by four amino acids was synthesized (JPT Peptide Technologies, Berlin, Germany) using the predicted amino acid sequences of the structural proteins of PRRSV-1 Olot/91 strain (GenBank Accession No. X92942.1) and the non-structural proteins of the closely related Lelystad strain (GenBank Accession No. AY588319.1) (27). Peptides were reconstituted and aliquots pooled to represent 19 proteins of PRRSV-1 as previously described (27). Antigenic M and NSP5 peptides were identified by screening peptides using a two-way matrix pooling system (38). Antigenic peptides with amino acid substitutions predicted from analyses of additional PRRSV strains were synthesized (JPT Peptide Technologies).

### Experimental PRRSV Infection of Pigs

All animal work was approved by the APHA Ethics Committee and conducted in accordance with the UK Animals (Scientific Procedures) Act 1986. An experimental infection and challenge study was carried out using 12-week-old, PCV-2 free, PRRSV antibody-negative Large White/Landrace cross-bred pigs. This experiment was designed to enable a comparison of T cell responses following primary infection to those boosted following secondary exposure. Animals were inoculated intranasally with 10^6^ TCID_50_ PRRSV-1 Olot/91 (*n* = 5) or 10^4^ TCID_50_ PRRSV-1 SU1-Bel (*n* = 5). Thirty-five days postinoculation, pigs were challenged with increased doses of the homologous virus, 10^7^ TCID_50_ PRRSV-1 Olot/91 and 10^5^ TCID_50_ PRRSV-1 SU1-Bel. Animals were monitored for clinical scores and rectal temperatures and blood samples were collected at intervals from 2 days postinfection (dpi) until the end of the study, 60 dpi, as described (5). Serum samples were stored at −80°C for subsequent analysis and heparinized blood was used for isolation of peripheral blood mononuclear cells (PBMCs).

### PRRSV Detection by Quantitative RT-PCR

Standard PRRSV-1 nucleoprotein gene (ORF7) RNA was prepared from Olot-91and SU1-Bel strains (5). RNA was isolated from serum using the QIAamp Viral RNA Mini Kit (Qiagen, Crawley, UK) and PRRSV RNA measured by quantitative real-time reverse transcription PCR (qRT-PCR) using the QIAGEN QuantiTect^®^ Probe RT-PCR kit (5).

### Detection of PRRSV-Neutralizing Antibody Responses

Porcine reproductive and respiratory syndrome virus nAb titers in serum samples were determined as described (6) with minor modifications. Serial twofold dilutions of heat-inactivated sera were incubated with 400 TCID_50_ of PRRSV for 1 h at 37°C. Neutralization of PRRSV-1 Olot/91 and SU1-Bel strains were assessed using MARC-145 cells (5 × 10^3^ cells/well) or PAMs (2 × 10^5^ cells/well), respectively. After 72 h incubation, infection was assessed by IPX and antibody titers calculated as log_2_ of the reciprocal serum dilution that fully neutralized viral replication in 50% of the wells.

### Assessment of PRRSV-Specific T Cell Responses in Peripheral Blood

Peripheral blood mononuclear cells were prepared from heparin blood as described and aliquots cryopreserved at a density of 1–2 × 10^7^ cells/ml in 10% DMSO in FBS (39). Fresh or previously cryopreserved PBMC were suspended in cRPMI and IFN-γ responses to antigenic stimulation assessed by ELISpot assay (27) or flow cytometry (38). For IFN-γ ELISpot assay, 5 × 10^5^ cells in 100 μl were added to wells of a precoated and blocked ELISpot plate (96-well Multiscreen-IP Filter Plates; Millipore, Watford, UK) and for flow cytometry 1 × 10^6^ cells in 50 μl were added to wells of a 96-well round-bottom tissue culture plate (Costar, Fisher Scientific, Loughborough, UK). Cells were stimulated with an equal volume of PRRSV-1 at a multiplicity of infection MOI = 0.1, or peptides at a final concentration of 1 μg/ml. Negative controls for peptide and virus stimulation were cells incubated in cRPMI alone or with mock-virus supernatants, respectively. For flow cytometry, PBMC were incubated at 37°C for 2 (peptide-stimulation) or 14–16 (virus-stimulation) hours and then Brefeldin A (GolgiPlug, BD Biosciences, Oxford, UK) was added at 1 μl/well and cells were further incubated for a further 16–18 or 6 h following stimulation with peptide or virus, respectively. In defined experiments to assess cytotoxic degranulation, CD107a-FITC or IgG1 isotype control-FITC mAbs (both AbD Serotec, Oxford, UK; 10 μl/well) and Monensin (Golgi Stop, BD Biosciences; 0.67 μl/ml) were added in conjunction with Brefeldin A (38). After incubation, PBMC were surface stained with the following directly conjugated mAbs: CD4-PerCP-Cy5.5 (clone 74-12-4, BD Biosciences), CD8α-PE (clone 76-2-11, BD Biosciences), and the Near Infra-Red Fixable Live/Dead Viability Dye (Life Technologies) or the Zombie Near Infra-Red Fixable Viability Kit (BioLegend, London, UK) (39). In defined experiments, CD44-eFluor450 (clone IM7, eBioscience, Hatfield, UK) or one of the following mAbs conjugated to Alexa Fluor^®^ 488 using Zenon Mouse IgG Labeling Kits (Life Technologies): CD62L (clone CC32, AbD Serotec), CD27 (clone b30c7, kindly provided by Dr. Wilhelm Gerner, University of Veterinary Medicine, Vienna, Austria) (40), and CD25 (clone K231.3B2, AbD Serotec) were additionally included in the surface stain. Following fixation and permeabilization, PBMC were stained with IFN-γ-Alexa Fluor 647 (clone CC302, AbD Serotec) and TNF-α-Brilliant Violet 421 (clone MAb11, Biolegend) or the respective isotype controls (Alexa Fluor 647 mouse IgG1 isotype control, AbD Serotec, and Brilliant Violet 421 mouse IgG1 isotype control, Biolegend) and analyzed using a MACSQuant Analyzer (Miltenyi Biotec, Bisley, UK) or a CyAn ADP flow cytometer (Beckman Coulter, High Wycombe, UK) (39). Live singlet CD4^+^CD8^int^ (CD4) and CD4^−^CD8^high^ (CD8) T cell populations (41) were gated upon and IFN-γ^+^ cells within these populations analyzed for coexpression of cell surface markers or TNF-α. Gates were set based on the corresponding isotype control and the number of singlet live lymphocytes acquired for analysis ranged from 200,000 to 400,000. In all instances, cell viability was confirmed to be >60%.

### MHC Haplotype Determination by Low-Resolution PCR-Based Analysis

Pigs were genotyped for their swine leukocyte antigen (SLA) class I and II haplotypes by running low-resolution PCR-screening assays (PCR-SSP) on PBMC-derived genomic DNA as previously described (42).

### Sequence Analysis of PRRSV

The predicted amino acid sequences of M (from 64 PRRSV-1 and 31 PRRSV-2 strains) and NSP5 (from 19 PRRSV-1 and 36 PRRSV-2 strains) proteins were aligned using the Clustal W algorithm on MegAlign (DNAStar Lasergene 9 Core Suite, Madison, WI, USA). The PRRSV-1 strains jointly analyzed for all three T cell antigenic regions were as shown in Figure 6B, with the GenBank accession numbers shown. The consensus sequence of the identified T cell antigenic regions from the PRRSV-2 strains examined was determined from the ClustalW alignment.

### Statistical Analysis

GraphPad Prism 6.01 (GraphPad Software, La Jolla, CA, USA) was used for graphical and statistical analysis of data sets. A one-way or two-way analysis of variance (ANOVA) was employed to analyze fixed effects on different traits with *post hoc* tests as detailed in the figure legends. A *p*-value <0.05 was considered statistically significant.

## Results

### Experimental Infection and Subsequent Homologous Challenge with PRRSV-1 Olot/91 or SU1-Bel and Association with Neutralizing Antibody and T Cell IFN-γ Responses

**Figure 1:**
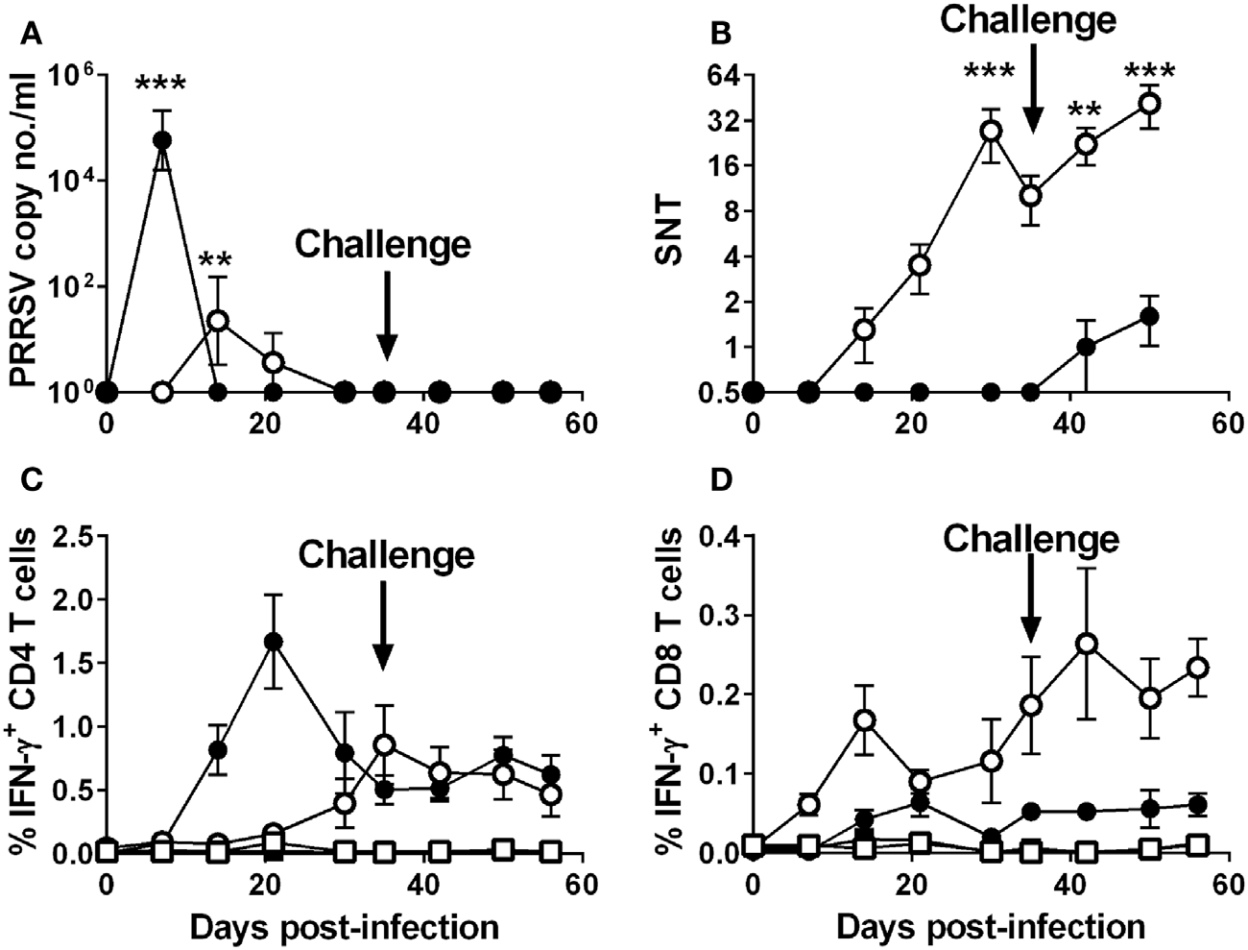
**Outcome of infection and subsequent challenge infection with PRRSV-1 strains and association with virus-specific immune responses**. Pigs were experimentally infected intranasally with either an attenuated PRRSV-1 strain (Olot/91; open symbols; *n* = 5) or a virulent sub-genotype 3 strain (SU1-Bel; closed symbols; *n* = 5) strains on day 0 and day 35 postinfection and PRRSV genome copy numbers in serum assessed **(A)**. Serum neutralizing antibody titers (SNT) were assessed against PRRSV Olot/91 infectivity *in vitro* **(B)**. IFN-γ expression by CD4^+^CD8α^low^ (CD4) and CD4^−^CD8α^high^ (CD8) T cells was assessed by flow cytometry following mock (square symbols) or homologous virus (circle symbols) stimulation [**(C,D)** respectively]. Results are expressed as the mean data for each group, error bars represent the SEM. For RT-qPCR data and VNTs, values were compared to the corresponding value on 0 dpi whereas for T cell IFN-γ data from virus-stimulated cultures were compared against the corresponding mock stimulated culture for each time point using a two-way analysis of variance (ANOVA) followed by a Tukey’s multiple comparison test; ***p < 0.001, **p < 0.01.

Groups of five pigs were inoculated intranasally with 10^6^ TCID_50_ of the attenuated PRRSV-1 subtype 1 strain Olot/91 or with a lower dose of 10^4^ TCID_50_ of the divergent virulent subtype 3 PRRSV strain with the aim of infecting animals but with a reduced likelihood of severe clinical disease. On day 35 postinfection, all animals were challenged by inoculation of the 10-fold higher dose of homologous virus than was used in the primary infection. As predicted, both groups showed mild clinical scores throughout the experiment, with the SU1-Bel group displaying several days (2 and 5–11 dpi) of elevated rectal temperatures following the primary inoculation (data not shown). Following the challenge at 35 dpi, there was no increase in clinical scores in the Olot/91 group and only mild hyperthermia observed on 37 and 38 dpi in the SU1-Bel group (data not shown). Quantitative RT-PCR analyses of serum samples showed a peak in PRRSV copy numbers in the Olot/91 group on 14 dpi (*p* < 0.01) that resolved completely by 30 dpi (Figure 1A). Statistically significant PRRSV copy numbers were seen on 7 dpi in the SU1-Bel group (*p* < 0.01), but these were rapidly cleared by 14 dpi. There was also no detectable viral RNA upon challenge, which taken together with the clinical scores suggests that the initial infection afforded a high degree of protection against reinfection.

In the PRRSV-1 Olot/91 infected group, Olot/91 neutralizing Abs were detectable from 14 dpi and levels steadily increased to a statistically significant peak at 30 dpi (*p* < 0.01), upon which levels began to wane (Figure 1B); however, there appeared to be a boosting effect upon challenge at 35 dpi. In the PRRSV-1 SU1-Bel-infected group, Olot/91 neutralizing Abs were not measurable until after the challenge (42 dpi, Figure 1B). SU1-Bel neutralizing Abs were not detected in the sera of animals from either infected group (data not shown). Assessment of PRRSV-specific T cell IFN-γ responses was conducted by flow cytometry following *ex vivo* stimulation of PBMC with the homologous virus. Both groups showed a higher magnitude of IFN-γ expressing CD4 (CD4^+^CD8α^low^) T cells than CD8 (CD4^−^CD8α^high^) T cells in response to homologous virus stimulation (Figures 1C,D). The Olot/91 group showed an increase in PRRSV-stimulated CD4 T cell responses from 21 dpi that were statistically significant from mock stimulation on 35 dpi and onward (*p* < 0.05). The SU1-Bel group displayed a higher overall magnitude of PRRSV-specific CD4 T cell responses, which were statistically significant from mock stimulation on 14 dpi and onward with a peak at 21 dpi (*p* < 0.05). PRRSV-specific CD8 T cell IFN-γ responses were observed at a much lower frequency compared to CD4 T cell responses. CD8 T cell responses were statistically significant compared to mock stimulation in the Olot/91 group on 14 dpi and from 30 dpi onward, and there were no statistically significant increases in PRRSV-specific CD8 T cell responses in the SU1-Bel group. No significant IFN-γ responses were detected CD4^+^CD8α^−^ or CD4^−^CD8α^−/low^ T cell populations (data not shown).

### Screening of PRRSV-1 Proteome-Wide Synthetic Peptide Library to Identify T Cell Antigens

**Figure 2:**
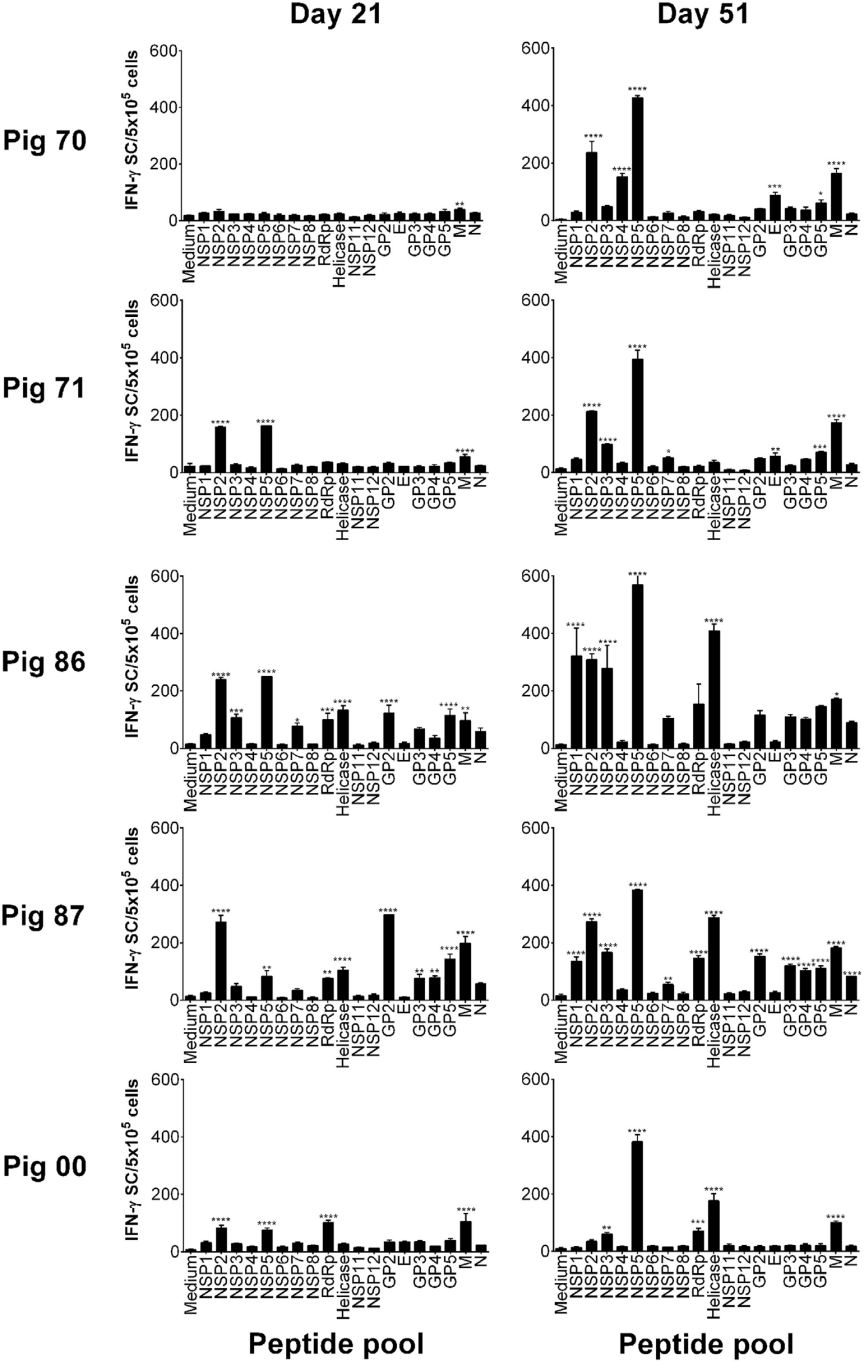
**Recognition of PRRSV-1 proteins by T cells from pigs experimentally infected with the Olot/91 strain**. PBMC from pigs experimentally infected with PRRSV-1 Olot/91 (*n* = 5) were isolated on day 21 and day 51 postinfection (16 days post-challenge), and stimulated *in vitro* with synthetic peptides pooled to represent 19 PRRSV-1 proteins. IFN-γ secreting cells were enumerated by ELISpot assay. Data are presented as IFN-γ spot forming cells (SFC)/5 × 10^5^ PBMC (triplicate cultures) for each animal and error bars show the SEM. Values for each peptide pool-stimulated condition were compared to the corresponding unstimulated (medium) control using a one-way ANOVA followed by a Dunnett’s multiple comparison test; ***p < 0.001, **p < 0.01, and *p < 0.05.

**Figure 3:**
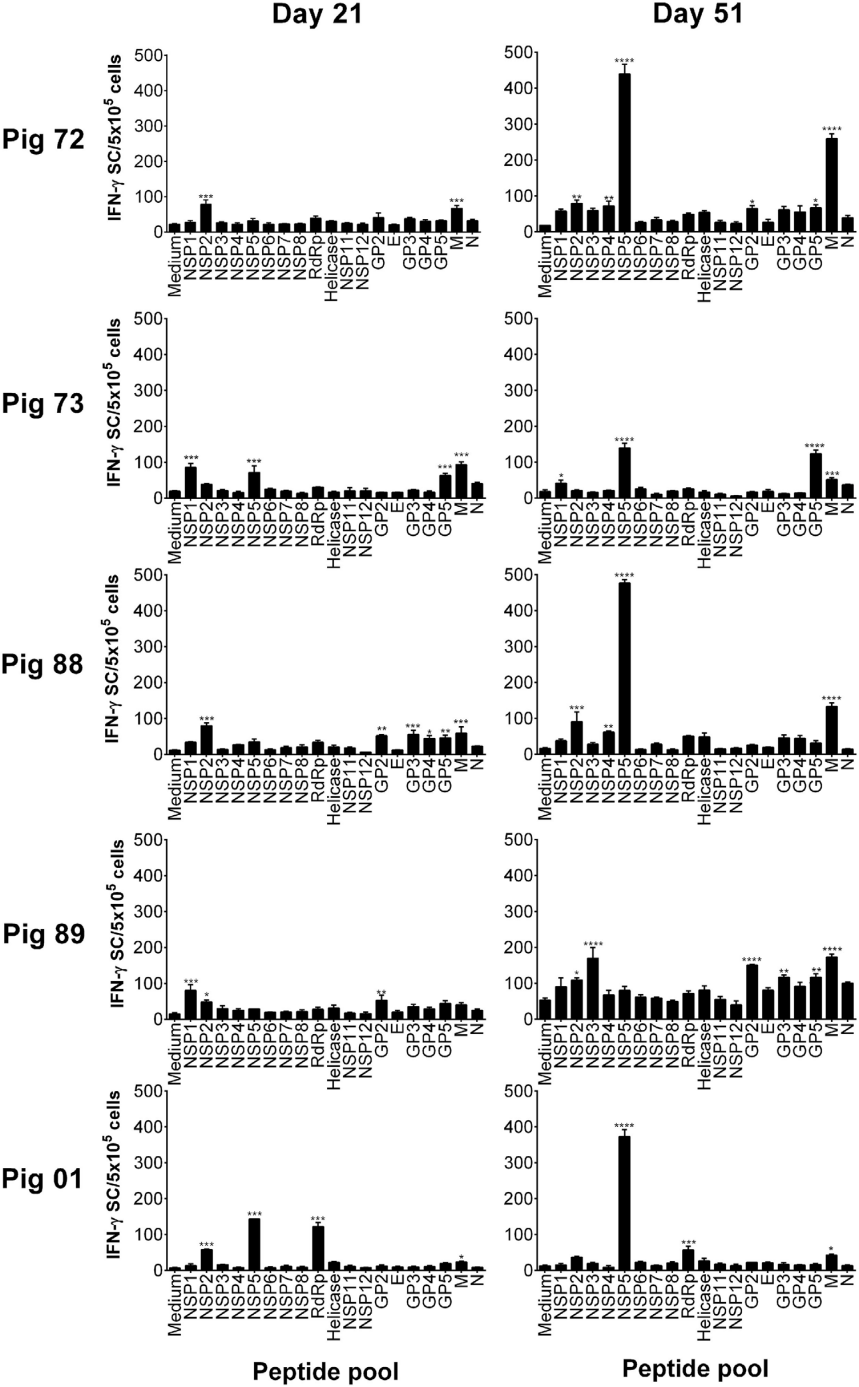
**Recognition of PRRSV-1 proteins by T cells from pigs experimentally infected with the SU1-Bel strain**. PBMC from pigs experimentally infected with PRRSV-1 SU1-Bel (*n* = 5) were isolated on day 21 and day 51 postinfection (16 days post-challenge), and stimulated *in vitro* with synthetic peptides pooled to represent 19 PRRSV-1 proteins. IFN-γ secreting cells were enumerated by ELISpot assay. Data are presented as IFN-γ spot forming cells (SFC)/5 × 10^5^ PBMC (triplicate cultures) for each animal and error bars show the SEM. Values for each peptide pool-stimulated condition were compared to the corresponding unstimulated (medium) control using a one-way ANOVA followed by a Dunnett’s multiple comparison test; ***p < 0.001, **p < 0.01, and *p < 0.05.

Peptides were pooled to represent PRRSV-1 proteins and recognition by PBMC assessed using an *ex vivo* IFN-γ ELISpot assay 21 days after infection (21 dpi) and 16 days after challenge (51 dpi) for both the Olot/91 (Figure 2) and SU1-Bel (Figure 3) groups. Similar to our earlier study (27), animals mounted responses to a range of PRRSV-1 peptide pools. Pigs in the Olot/91 group displayed a greater magnitude of response to peptide pools after challenge as well as an increase in the number of pigs mounting significant responses to particular pools. The most prominent response in terms of the proportion of animals mounting a significant response was to the M peptide pool, to which all pigs showed a statistically significant response at both time points (Figure 2). Peptides representing NSP5 also induced significant IFN-γ responses, with 4/5 of pigs responding after infection and all pigs responding after challenge. Similar to the Olot/91 group, the SU1-Bel group also displayed greater T cell IFN-γ responses after challenge (Figure 3). The most striking observation was the magnitude of the IFN-γ response to the NSP5 peptide pool after challenge in 4/5 animals compared to 2/5 after infection. Interestingly, pig 88 did not mount a significant response to NSP5 after infection but the response after challenge had the greatest magnitude of all. Significant responses were also observed against the M peptide pool with 4/5 responders after infection and 5/5 responders after challenge. Thus, in both groups, M and NSP5 peptide pools were the most dominant antigens in terms of frequency of responder animals and the magnitude of the response and were selected for further study.

### Characterization of the Kinetics and Phenotype of PRRSV-1 M- and NSP5-Specific T Cell Responses

**Figure 4:**
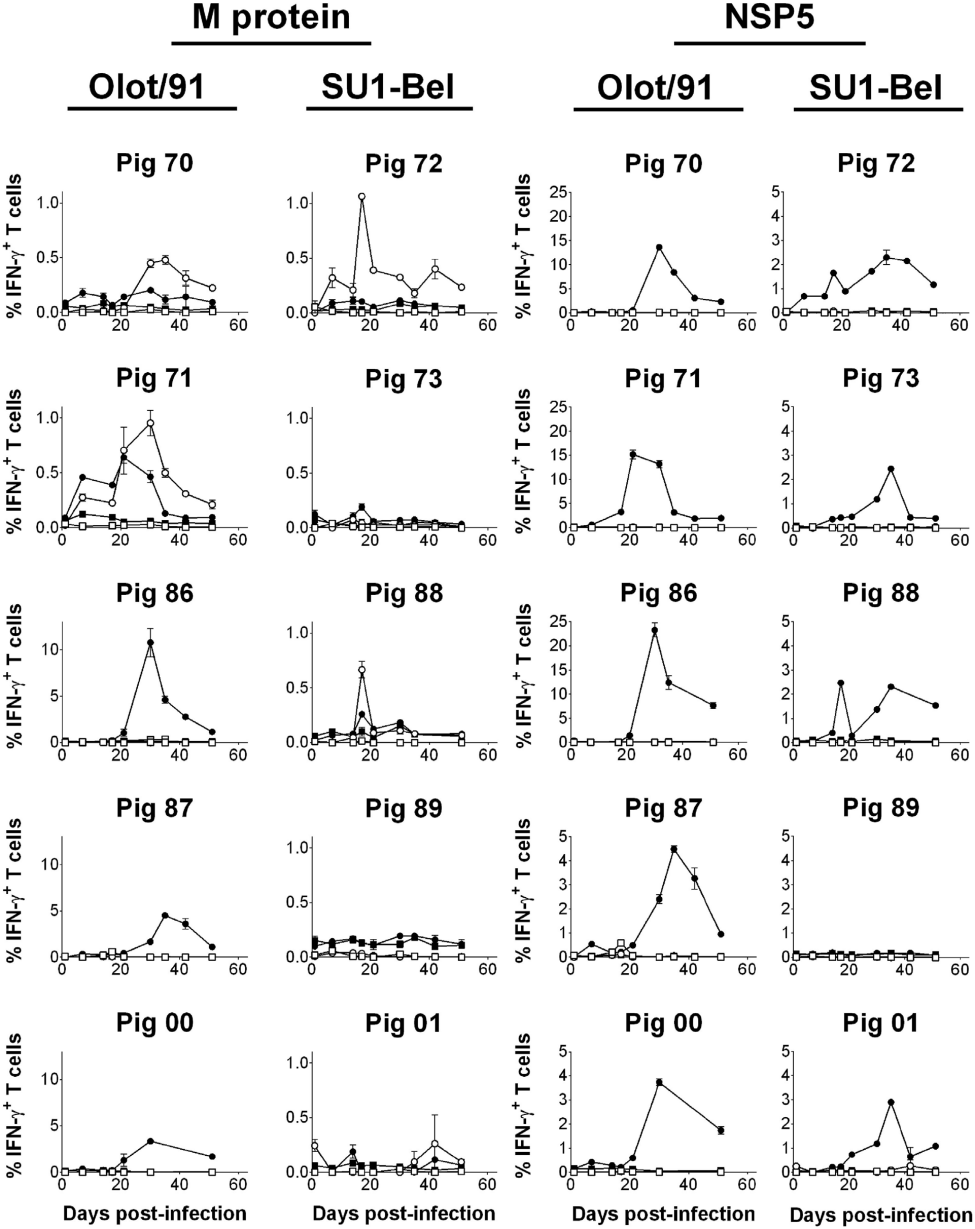
**Assessment of NSP5- and M-specific IFN–γ T cell responses over the course of infection and challenge with PRRSV-1 Olot/91 and SU1-Bel strains**. Previously cryopreserved PBMC were stimulated *ex vivo* with synthetic peptide pools representing M or NSP5 protein or left unstimulated. IFN-γ expression by CD4^+^CD8α^low^ (CD4; open symbols) and CD4^−^CD8α^high^ (CD8; closed symbols) T cells from unstimulated (square symbols) or peptide pool-stimulated cultures (circle symbols) was assessed by flow cytometry. The mean % of IFN-γ^+^ T cells from duplicate cultures are presented for each animal and error bars show the SEM. Data from peptide pool-stimulated cultures were compared against the corresponding unstimulated culture using a two-way ANOVA followed by a Tukey’s multiple comparison test.

The T cell responses to peptide pools representing M and NSP5 proteins were measured longitudinally over the time course of infection and challenge using previously cryopreserved PBMC and flow cytometry. The IFN-γ response to the M protein differed significantly between individual animals both in terms of magnitude and phenotype (Figure 4). The frequencies of M-specific IFN-γ secreting CD8 T cells observed in pigs 86, 87, and 00, all infected with Olot/91, were of an order of magnitude greater than the other animals and are therefore presented with a different *y*-axis scaling. Compared to unstimulated controls, these three animals all mounted a significant CD8 T cell response to the M peptide pool, as did the remaining animals in the Olot/91 group, pigs 71 and 70 (*p* < 0.01). Pigs 70 and 71 also mounted a CD4 T cell response, which was greater in magnitude than their CD8 T cell response (*p* < 0.01). Within the SU1-Bel group, pigs 72 and 88 also mounted a significant CD4 (CD4^+^CD8α^low^) T cell response to the M peptide pool (*p* < 0.01). Pigs 73, 89, and 01 did not mount a significant response to the M peptides at any time point tested with the preserved cells. The earliest statistically significant T cell response was seen on 7 dpi in pigs 71 (CD8) and 72 (CD4) and significant responses continued until 51 dpi (pigs 70 and 72 – CD4; pigs 87 and 00 – CD8).

As with the response to the M peptides, there was a variation in the magnitude and kinetics of T cell IFN-γ responses to the NSP5 peptides between individual animals (Figure 4). Compared to M-specific responses, the IFN-γ response to NSP5 was in the majority of animals greater and in all cases exclusively from CD8 T cells. As was seen in the IFN-γ ELISpot assay data (Figures 2 and 3), all pigs responded to NSP5 with the exception of pig 89 (*p* < 0.01). Pigs 70, 71, and 86 (all infected with Olot/91) presented with exceptionally strong responses to NSP5, peaking at a frequency of >10% CD8 T cells expressing IFN-γ in response to NSP5 stimulation, and therefore, these animals are shown with a separate *y*-axis scaling. Responses tended to peak between days 21 and 35 after infection, and there was no clear evidence of boosting after challenge.

### Characterization of the Activation/Memory Phenotype and Polyfunctionality of PRRSV-1 M- and NSP5-Specific T Cell Responses

**Figure 5:**
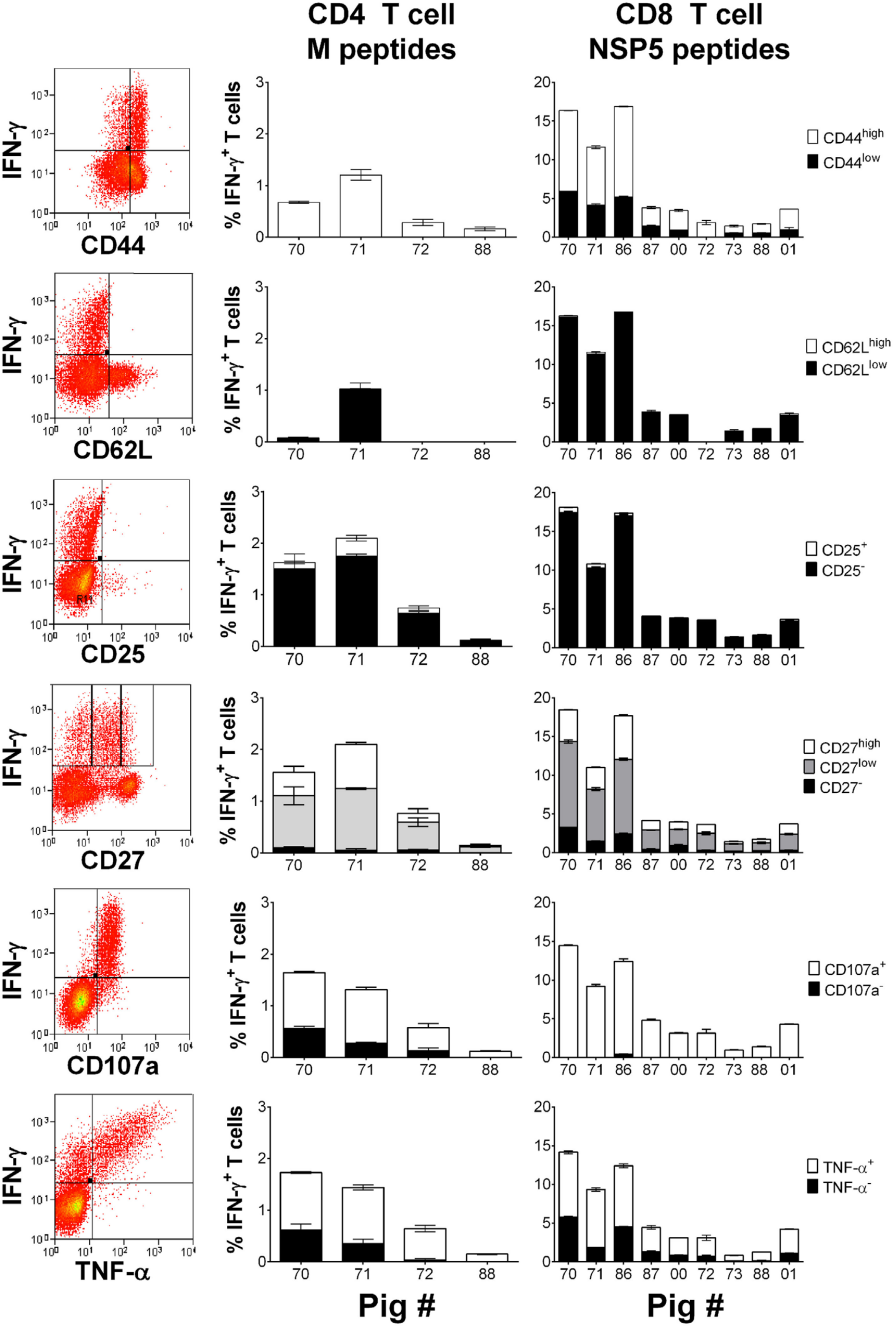
**Assessment of the phenotype and polyfunctionality of PRRSV-1 NSP5-specific CD8 T cells**. Previously cryopreserved PBMC from identified T cell responder pigs on day 30 postinfection were stimulated with synthetic peptide pools representing M or NSP5 proteins or left unstimulated. The expression of CD44, CD62L, CD25, CD27, surface CD107a, and TNF-α by IFN-γ^+^ CD4 T cells in response to M peptides and CD8 T cells in response to NSP5 peptides were assessed by flow cytometry as shown by representative dot plots. The mean % of unstimulated-corrected data from duplicate cultures are presented for individual animals and error bars show the SEM.

Peripheral blood mononuclear cells previously isolated from selected responder pigs from each infection group on 30 dpi were stimulated with either M or NSP5 peptide pools to determine the expression of markers of activation, memory, or functionality on antigen-specific T cells. Stimulated cells were stained to additionally assess the expression of CD44, CD62L, CD25, CD27, the cytotoxic degranulation maker CD107a, and TNF-α by IFN-γ^+^ CD4 and CD8 T cells (Figure 5). The results showed that the majority of IFN-γ^+^ NSP5-specific CD8 T cells and M-specific CD4 T cells expressed high levels of CD44 (*p* < 0.01), low levels of CD62L (*p* < 0.01), and lacked expression of CD25 (*p* < 0.01). IFN-γ expressing M-specific CD4 and NSP5-specific CD8 T cells displayed variable levels of CD27, but the greatest number were classified as CD27^low^ (*p* < 0.01) followed by CD27^high^ (*p* < 0.05). Almost all IFN-γ^+^ NSP5-specific CD8 T cells and surprisingly the majority of M-specific CD4 T cells had mobilized CD107a to their surface (*p* < 0.01). The majority of antigen-specific cells coexpressed IFN-γ and TNF-α (*p* < 0.01), with the absence of a significant proportion of antigen-specific cells expressing TNF-α alone (data not shown).

### Mapping of Responses on PRRSV-1 M and NSP5 Proteins and Assessment of the Effect of Antigenic Polymorphism and MHC Haplotype Expression

Identification of the antigenic peptide targets of M- and NSP5-specific T cell responses was assessed using a two-way matrix pooling system to screen the individual 15mers. Putative antigenic peptides were identified from the matrix pool screens then screened individually (Data Sheet S1 in Supplementary Material). Pigs 70 and 71, from the Olot/91 group, and pigs 72 and 88, from the SU1-Bel group, presented significant CD4 T cell responses to M peptide #8 (M_29–43_; MIYALKVSRGRLLGL) and pig 71 also showed reactivity to the overlapping peptide #9 (M_33–47_; LKVSRGRLLGLLHIL). CD8 T cells from pigs 70, 71 (both Olot/91 group), and 88 (SU1-Bel group) showed a statistically significant response to NSP5 peptide #4 (NSP5_13–27_; FLLWRMMGHAWTPIV). In addition, these pigs, as well as pigs 72, 73, and 01 (all SU1-Bel group), showed a significant response to overlapping NSP5 peptides #39 and #40 (NSP5_153–167_ and NSP5_156–170_) with the consensus sequence NSP5_156–167_ (DGSFSSAFFLRY). Pigs 86, 87, and 00 responded to a second set of overlapping NSP5 peptides #37 and #38 (NSP5_145–159_ and NSP5_149–163_) with the consensus sequence NSP5_149–159_ (LHNMLVGDGSF). A summary of the identified antigenic regions are shown in Table 1.

**Table 1:** **Porcine MHC (SLA) class I and II low-resolution (Lr) haplotypes of PRRSV-infected pigs and T cell reactivity against identified antigenic peptides**.

Group	Pig	SLA-I Lr haplotype	CD8 T cell antigenic peptide			SLA-II Lr haplotype	CD4 T cell antigenic peptide
Olot/91	70	22.0/35.0	NSP5_13–27_		NSP5_156–167_	0.01/0.15b	M_29–43_
	71	22.0/35.0	NSP5_13–27_		NSP5_156–167_	0.01/0.15b	M_29–43_
	86	35.0/38.0		NSP5_149–159_		0.11/0.15b	
	87	35.0/38.0		NSP5_149–159_		0.11/0.15b	
	00	43.0/ND^a^		NSP5_149–159_		0.01/0.02	
SU1-Bel	72	22.0/35.0			NSP5_156–167_	0.01/0.15b	M_29–43_
	73	22.0/62.0			NSP5_156–167_	0.01/0.11	
	88	22.0/35.0	NSP5_13–27_		NSP5_156–167_	0.01/0.15b	M_29–43_
	89	35.0/38.0				0.11/0.15b	
	01	04.0/22.0			NSP5_156–167_	0.01/0.02	

*^a^ND, not determined*.

Animals were MHC typed to assess whether peptide-specific T cell responses could be attributed to specific MHC class I and II haplotypes (Table 1). With regards to MHC class I, pigs 70, 71, 72, and 88 were haploidentical heterozygotes, expressing haplotypes Lr-22.0 and 35.0. Pigs 86, 87, and 89 also shared the haplotype Lr-35.0 with these animals, as well as haplotype Lr-38.0 between themselves. This may suggest that the antigenic region NSP5_156–167_ recognized by pigs 70, 71, 72, 73, 88, and 01 was restricted by the SLA-I haplotype Lr-22.0. The antigenic region NSP5_145–159_ appeared to be restricted by more than one haplotype since pig 00 did not share either of its haplotypes with any of the other responders. It could be speculated that this region is potentially restricted by Lr-38.0 (taking into account the non-responding pig 89) and Lr-35.0 in the absence of Lr-22.0. The antigenic region NSP5_13–27_ could similarly be restricted by multiple haplotypes. The pigs that responded to the CD4 T cell antigenic peptide M_29–43_ (pigs 70, 71, 72, and 88) were haploidentical, sharing both SLA-II haplotypes Lr-0.01 and 0.15b, this combination appearing exclusively in these animals suggesting restriction by one of these two haplotypes.

**Figure 6:**
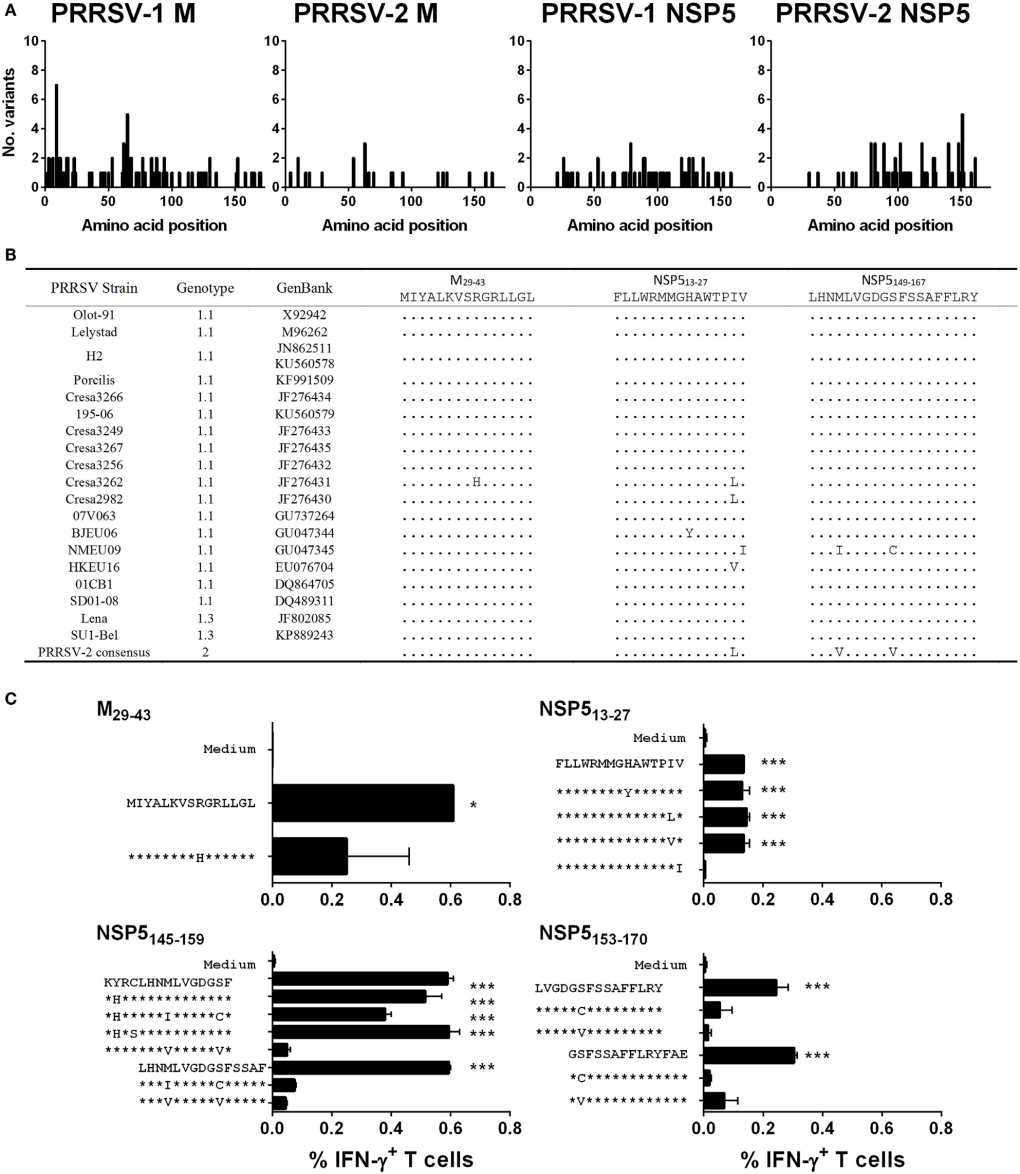
**Assessment of the conservation of identified T cell antigens M and NSP5 among PRRSV strains and assessment of T cell reactivity against variant peptides**. The complete predicted amino acid sequences of M (from 64 PRRSV-1 and 31 PRRSV-2 strains) and NSP5 (from 19 PRRSV-1 and 36 PRRSV-2 strains) were aligned and the number of different amino acid variants at each residue plotted **(A)**. The predicted amino acid sequences of identified antigenic regions, M_29-43_, NSP5_13-27_, and NSP5_149-167_, were compared among the panel of 19 PRRSV-1 strains, for which both M and NSP5 sequence data were available, and a consensus sequence based on available PRRSV-2 strains **(B)**. Based on the observed amino acid substitutions, variant peptides were used to stimulate PBMC from pigs 71 and 86 **(C)**. IFN-γ expression by CD4 T cells to M_29-43_ (pig 71) and CD8 T cells to NSP5_13-27_ (pig 71), NSP5_145-159_ (pig 86), and NSP5_153-170_ (pig 71) peptides were assessed by flow cytometry. The mean% of unstimulated (medium) and peptide stimulated data from duplicate cultures are presented and error bars show the SEM. Values were compared to the unstimulated control using a one-way ANOVA followed by a Dunnett’s multiple comparison test; ***p < 0.001, **p < 0.01, and *p < 0.05.

The level of amino acid sequence conservation of antigenic peptides both within genotype 1 PRRSV strains and between genotypes was investigated to further evaluate the identified antigens. The complete predicted amino acid sequences of M from 64 PRRSV-1 and 31 PRRSV-2 strains and NSP5 from 19 PRRSV-1 and 36 PRRSV-2 strains were aligned and the number of different amino acid variants at each residue plotted (Figure 6A). The results showed that both proteins were well conserved with either no or only a single amino acid substitution being observed for the majority of residues. There was no evidence of regions of hypervariability in either of the antigens. The predicted amino acid sequences of identified antigenic regions, M_29–43_, NSP5_13–27_, and NSP5_149–167_, were compared among the panel of 19 PRRSV-1 strains, for which both M and NSP5 sequence data were available, and a consensus sequence based on available PRRSV-2 strains (Figure 6B). All antigenic sequences were conserved between PRRSV-1 Olot/91 and the SU1-Bel strain, which explained the observed conservation in the specificity of responses across the infection groups. Identified variant sequences were synthesized and tested for their ability to induce T cell IFN-γ responses from representative pigs 86 and 71 (Figure 6C). The CD4 T cell antigenic region of M was well conserved with only one variant, a related substitution of a histidine for an arginine at position 37, which still stimulated a CD4 T cell response (although not deemed statistically significant). The NSP5_13–27_ antigenic region had four potential amino acid substitutions, all but one of which still induced a statistically significant CD8 T cell IFN-γ response. The individual 15mers that made up NSP5_145–159_ possessed substitutions at four positions; however, all but one of the six variants induced a statistically significant IFN-γ response (although four of these lay outside the consensus sequence and therefore probably had no effect on the antigenic region). While there were only two amino acid substitutions in the NSP5_153–170_ region, these removed the peptides ability to stimulate a CD8 T cell IFN-γ response.

## Discussion

IFN-γ secreting T cell responses appear important, if not essential, for resolution of PRRSV infection (15). The present study showed, at least in the context of PRRSV-1 SU1-Bel infection, that viremia was cleared and protection against secondary infection occurred in the absence of measurable nAbs but not T cell IFN-γ responses, suggesting the latter response could be sufficient to protect against PRRSV infection. These data support our earlier study of the immune responses induced by SU1-Bel (5). The ability of PRRSV infection to prime both CD4 and CD8 T cells was in accordance with the literature, also agreeing with the stronger observed CD4 T cell response following *in vitro* stimulation with PRRSV (43). However, comparison with the frequencies of PRRSV peptide-specific CD4 and CD8 T cells revealed that the assessment of PRRSV-specific CD8 T cell responses by stimulation with live virus greatly underestimated the true response. It may be speculated that a low level of productive PRRSV infection occurs within PBMC, and this limits the availability of NSPs and/or trafficking of PRRSV peptides into the “direct” or cytosolic MHC class I processing and presentation pathway resulting in suboptimal CD8 T cell stimulation.

Strong T cell responses were directed against the M and NSP5 proteins in pigs infected with both Olot/91 and SU1-Bel strains. These proteins have been identified previously as bearing T cell epitopes (27, 32, 35, 37), but the phenotype of the responding T cells had, in most instances, not been defined. Indeed, the identity of NSP5 peptides that induce IFN-γ has only been reported in one other study to date, and none of the antigenic peptides matched those identified here (37). A T cell antigenic region of the M protein, “M6,” previously identified from a highly pathogenic PRRSV-2 strain (35) shows a significant overlap with the CD4 T cell antigenic peptide M_29–43_ identified here. The epitopes on each antigen that the individual pigs responded to appear dependent on the MHC haplotypes of the pigs rather than the virus strain they were infected with and certain antigenic regions could potentially be attributed to, in some instances, multiple haplotypes. In our previous evaluation of T cell responses using the PRRSV-1 peptide library (27), we observed significant M-specific responses from animals infected with Olot/91, SU1-Bel, and Lelystad strains, whereas NSP5-specific responses were only detectable within the SU1-Bel infected group, suggesting that the combination of infecting strain and MHC haplotype influences the antigen-specificity of T cell responses. The antigenic peptides in the present study were highly conserved among PRRSV-1 strains and in some cases with PRRSV-2 strains. Pigs could still respond to antigenic peptides with certain amino acid substitutions identified in divergent strains, for example in NSP5_153–170_, which is encouraging for vaccine applications when considering the diversity of PRRSV strains in the field.

The M protein was recognized by both memory CD4 (CD4^+^CD8α^low^) and CD8 (CD4^−^CD8^high^) T cells (41, 44), whereas the NSP5 was the target of a CD8 T cell response. A cytotoxic CD8 T cell response is important for the control of viral replication, especially in chronic or persistent viral infections, but CD4 T cells also play a crucial role in the induction of CD8 T cell responses. According to the current paradigm, two distinct subsets of memory T cell exist, effector memory (T_EM_) and central memory (T_CM_) (45). These can be distinguished by the lymph node homing markers CD62L and CCR7, with CD62L^+^CCR7^+^ and CD62L^−^CCR7^−^ cells representing the T_CM_ and T_EM_, respectively (46). T_EM_ cells are highly enriched at tissue effector sites including the lungs and mucosae, are polyfunctional expressing multiple effector cytokines (TNF-α and IFN-γ), and with relatively immediate cytotoxic potential. T_CM_ are instead primarily localized to central lymphoid tissue and are also relatively delayed in their effector response requiring *de novo* exposure to the invading pathogen before acquiring full effector function (45). Phenotyping of the IFN-γ expressing responder CD4 and CD8 T cells showed that the majority of cells expressed high levels of CD44, low levels of CD62L and CD27, lacked expression of CD25^−^, displayed a marker of cytotoxic degranulation (CD107a), and coexpressed TNF-α. Based on the studies in humans and mice, the high expression of cell adhesion molecule CD44 and low expression of CD62L putatively identifies these responding T cells as effector (T_EFF_) or T_EM_ cells. The majority of IFN-γ^+^ T cells expressed low levels of CD27, which may classify them as T_EM_ cells if orthologous to the human system (47, 48). The lack of coexpression of CD25 and IFN-γ on M- and NSP5-specific T cells further suggested a T_EM_ phenotype. These results make a further contribution to the growing body of data delineating porcine memory T cell subsets (44). Dual or polyfunctional T cells as defined by the simultaneous secretion of IFN-γ and TNF-α have been correlated with the enhanced quality and robustness of the T cell response (49) and the ability of the M and NSP5 proteins to stimulate these dual cytokine expressing cells further supports the use of these two antigens in future vaccine development. CD107a mobilization is considered a marker for cytotoxic degranulation (50) and the majority of the M- and NSP5-specific T cells were shown to express both IFN-γ and CD107a. CD107a expression has been described on antigen-specific porcine CD8 T cells previously (39). Its expression on porcine CD4 T cells was unexpected although CD4^+^CD8^+^ T cell cytotoxic activity has been described in the context of African swine fever infection (51), as well as in humans and mice (52).

In conclusion, this study has used an unbiased strategy to identify PRRSV-1 antigens. Our characterization of the PRRSV-1 M and NSP5 antigens and the specific polyfunctional T_EM_ responses strongly suggest their vaccine potential and provide a solid basis for their evaluation in efforts to develop a subunit-based next-generation PRRSV vaccine.

## Author Contributions

The study was conceived and experiments designed by HM, J-PF, GS, SS, FS, and SG. HM, MP, J-PF, LB, SH, LK, LL, SS, and SG performed the experiments. Data were analyzed and interpreted by HM, MP, J-PF, SH, and SG. While HM and SG drafted the paper, MP, J-PF, LB, SH, LK, LL, GS, SS, and FS all critically revised the manuscript for important intellectual content.

## Conflict of Interest Statement

The authors declare that the research was conducted in the absence of any commercial or financial relationships that could be construed as a potential conflict of interest.
