# Supplementary material for: The Non-structural Protein 5 and Matrix Protein Are Antigenic Targets of T Cell Immunity to Genotype 1 Porcine Reproductive and Respiratory Syndrome Viruses
Source: Front Immunol. 2016 Feb 16;7:40. doi: 10.3389/fimmu.2016.00040 (PMC4755262; doi:10.3389/fimmu.2016.00040)
Supplement: Supplementary file 1 [file datasheet_1.pdf]

A

	NSP5 Matrix Pools*					
NSP5 Matrix Pools	**A#**	**B#**	C	D	E	F
G	1	2	3	4	5	6
H	7	8	9	10	11	12
I	13	14	15	16	17	18
J	19	20	21	22	23	24
K	25	26	27	28	29	30
L	31	32	33	34	35	36
**M#**	**37**	**38**	39	40		

B

		CD4 T cell – M peptides		CD8 T cell – NSP5 peptides	
Group	Pig	Positive matrix pool	Corresponding peptides	Positive matrix pool	Corresponding peptides
Olot/91	70	A B C H	7 8 9	C D E F G M	3 4 5 6 39 40
	71	A B C H	7 8 9	C D E G M	3 4 5 39 40
	86	-	-	A B M	37 38
	87	-	-	A B M	37 38
	00	-	-	A B M	37 38
SU1-Bel	72	A B C H	7 8 9	C D G M	3 4 39 40
	73	-	-	C D M	39 40
	88	A B H	7 8	A C D G M	1 3 4 37 39 40
	89	-	-	-	-
	01	A B H	7 8	C D M	39 40

C

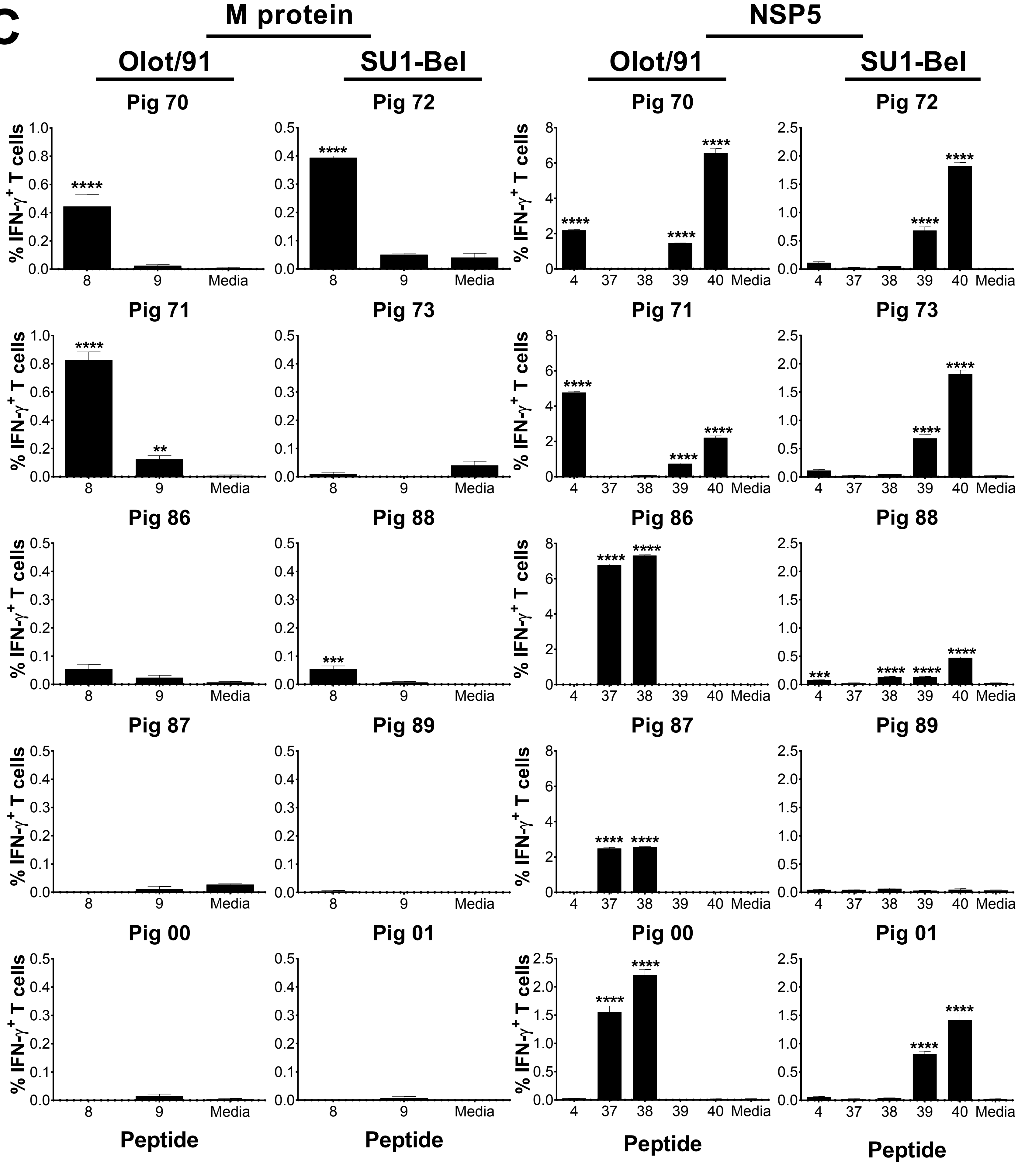

Supplementary Data Sheet 1. Identification of antigenic peptides within PRRSV-1 M and NSP5 peptide pools. Individual peptides representing PRRSV-1 M and NSP5 proteins were combined using a two-way matrix-pooling system as illustrated for NSP5 peptides (A): The 40 overlapping 15mer peptides (#1-40) representing NSP5 were pooled so that each peptide was uniquely represented in two matrix-pools (Pools A-M). #Matrix pools A, B and M stimulated CD8 T cell IFN-γ reactivity from pig 86 and so overlapping peptides #37 and #38 were identified for further testing. Screening of matrix pools identified a number of putative antigenic M and NSP5 peptides recognised by CD4 and CD8 T cells, respectively (B). T cell reactivity against selected putative antigenic peptides was assed using PBMC isolated from pigs on day 58 post-infection (C). IFN-γ expression by CD4 T cells to individual M peptides and CD8 T cells to individual NSP5 peptides were assessed by flow cytometry. The mean % of unstimulated (media) and peptide stimulated data from duplicate cultures are presented and error bars show the SEM. Values were compared to the unstimulated control using a one way ANOVA followed by a Dunnett's multiple comparisons test; **** $p<0.0001$, *** $p<0.001$, ** $p<0.01$.
